# Interferon-Beta-1b Induced Autoimmune Hemolytic Anemia in a Patient with MS: A Case Report

**Published:** 2011-03-01

**Authors:** M Saeedi, M Forughipour, P Sasannezhad, A Shoeibi

**Affiliations:** 1 Department of Neurology, Ghaem Hospital, Mashhad University of Medical Sciences, Mashhad, Iran

**Keywords:** Multiple sclerosis, Interferon beta, Haemolytic anemia

## Abstract

A 26-year-old lady with the diagnosis of multiple sclerosis who had received interferon beta1-b for eleven months was visited in MS clinic of our hospital because of icter and fatigue. Laboratory tests showed anemia, indirect hyperbillirubinemia, increased LDH, positive direct and indirect coomb’s tests, and increased reticulocyte count and percentage. Other causes of autoimmune hemolytic anemia (AHA) and pre-existing AHA in the patient were ruled out. After INF discontinuation, symptoms disappeared, hemoglobin increased, and indirect coomb’s test became negative. We concluded that autoimmune hemolytic anemia should be considered in all MS patients who receive interferon beta1-b and present with symptoms and signs of anemia.

## Introduction

The first treatment measure in multiple sclerosis (MS) approved by the FDA in 1993 was interferon beta1-b (INFβ1-b; betaseron).[1] The immunomodulatory effects of INFβ1-b may be the result of increased suppressor cell activation, inhibition of cytotoxic T-cells, cytokine changes and effects on the blood-brain barrier. While most patients with MS benefit from these immunomodulatory effects, some autoimmune disorders such as thyroid dysfunction and hepatitis may be induced or aggravated by this treatment.[2] A case of interferon induced autoimmune hemolytic anemia (AHA) is reported in this paper. To the best of our knowledge, this is the second case of betaseron induced autoimmune hemolytic anemia in patients with relapsing remitting MS (RRMS) in the world. The first case was reported by Alanoglu et al.[3]

## Case report

A 26-year-old lady with RRMS since 11 months ago who had been treated with betaseron (8 mIU every other day) was visited in MS clinic of our hospital affiliated to Mashahd University of Medical Sciences in Mashahad, Iran because of icter and fatigue from 3 days ago. Her disease had started from 13 months ago with left optic neuritis. The first attack had improved completely with high dose intravenous prednisolone (1 gr/day for 5 consecutive days). Two months later, a second attack occurred as spastic paraparesis. The patient received another 5-day intravenous corticosteroid pulse therapy and after complete recovery, INFβ1-b was started. The patient was doing well until she visited because of fatigue and icter. At this time, neurologic examination was normal. She had received no drug other than betaseron. The patient had no history of viral infection, fauvism, blood transfusion or additional drug intake. Physical examination showed pale conjunctiva and icteric sclera without organomegaly or lymphadenopathy. La ratory tests were as follows (normal ranges are in parenthesis): Total billirubin: 8.5 mg/dl (<1.1 mg/dl), direct billirubin: 0.4 mg/dl (<0.25 mg/dl), SGOT: 24 IU/L (up to 40), SGPT: 27 IU/L (up to 40), Alkaline phosphatise: 140 U/L (64-306), LDH: 819 IU/l (up to 480), HBsAg: Negative, Anti HCV Ab (IgM and IgG): Negative, indirect coomb’s test: positive, Direct coomb’s test: Positive, ANA: Negative, Anti-DNA (ds): Negative, rheumatoid factor: Negative, blood iron profile: Normal, reticulocyte: 2.3% corrected (≤2%), serum protein and immunoelectrophoresis: Normal, U/A: Normal, WBC: 8400 (Neut: 66%, lym: 30%, Mono: 2%, Eos: 2%), RBC: 3.44 × 10(6), Hb: 10.3 g/dl, Hct: 32.5, MCV: 94.5 fL, MCH: 29.9 pg, MCHC: 31.7 g/dl, platelet count: 275000 and erythrocyte sedimentation rate: 20 mm/h. The red blood cell morphology of the peripheral blood showed spherocytosis, mild macrocytosis, and polychromatophilia. Ultrasonography of the liver, spleen, portal vein and gall bladder was normal.

Other causes of AHA were ruled out or the patient. Medical records of the patient were reviewed comprehensively and pre-existing AHA was ruled out. INFβ1-b was discontinued and the patient was observed carefully for 4 weeks. No additional drug was started for the treatment of patient’s anemia. The patient became asymptomatic and icter disappeared about 3 weeks after drug discontinuation. Indirect coomb’s test became negative, direct coomb’s test titer decreased and hemoglobin level increased to 13.4 g/dl. Since that time -6 months ago- the patient is doing well without receiving any medication. Fortunately, the patient had no relapse of MS and no disease modifying treatment measure was needed.

## Discussion

MS, similar to the other immune related disorders, may be associated with some autoimmune diseases such as Hashimoto’s thyroiditis, Graves’ disease, rheumatoid arthritis, systemic lupus erythematosus, and insulin-dependent diabetes mellitus.[4][5] Interferon may induce autoimmune disorders. Some autoimmune disorders including thyroiditis, thrombocytopenia, anemia, hepatitis, and diabetes has been reported in association with INF-α therapy for some infective (e.g., chronic hepatits C) and hematological disorders.[6][7] The immunostimulant effects of INF may be responsible for these autoimmune disorders. INF may increase the major histocompatibility complex (MHC) molecule expression, enhance immunoglobulin (Ig) and inflammatory cytokine release, and inhibit activated T cell apoptosis.[3]

One of the first reports of INF induced AHA is the Akard’s study which reported AHA due to INF-α.[8] Since that time, Several case reports reflected the AHA in hematologic or infectious disorders under INF-α therapy Kazuta et al. reported INF-β induced AHA for the first time.[9] His patient was on INF-β for the treatment of chronic hepatitis C. Riechmann et al. reported a suspected INFβ1a induced AHA case who was treated with the diagnosis of MS by INFβ1a for 2.5 years. When INFβ1a was restarted for the patient, hematologic abnormalities reappeared.[10]

By the best of our knowledge, this is the second report of INFβ1b induced AHA in MS patients. Alanoglu et al. reported the first case of INFβ1b induced AHA in MS patients.[3] The patient was a 31- year old male with MS who had received INFβ1b for two years. They started prednisone (1 mg/kg) with gradual tapering in several weeks in addition to INF discontinuation. Our patient had received INFβ1b (betaseron) for 11 months. AHA was confirmed by anemia, indirect hyperbillirubinemia, increased LDH, positive direct and indirect coomb’s tests, and increased reticulocyte percentage. Other causes of AHA and pre-existing AHA in the patient were ruled out. Symptoms disappeared a indirect coomb’s test became negative after one month of INF discontinuation without corticosteroid treatment. In conclusion, if an unexplained drop in hematocrit occurs in a MS patient who receives INFβ1b, AHA should be considered and be ruled out. Discontinuation of INFβ1b seems reasonable. Fortunately, our patient had no MS relapse after discontinuation of INF. Whether or not INF can be restarted needs more clinical experience.
